# Evaluation of *CERS2* Gene as a Potential Biomarker for Bladder Cancer

**DOI:** 10.1155/2019/3875147

**Published:** 2019-09-15

**Authors:** Ahmed Faris Aldoghachi, Aminuddin Baharudin, Umar Ahmad, Soon Choy Chan, Teng Aik Ong, Rosna Yunus, Azad Hassan Razack, Khatijah Yusoff, Abhi Veerakumarasivam

**Affiliations:** ^1^Medical Genetics Laboratory, Faculty of Medicine and Health Sciences, Universiti Putra Malaysia, Selangor Darul Ehsan, Malaysia; ^2^Perdana University School of Foundation Studies, Perdana University, Selangor Darul Ehsan, Malaysia; ^3^Department of Surgery, Faculty of Medicine, University of Malaya, Wilayah Persekutuan, Kuala Lumpur, Malaysia; ^4^Histopathology Unit, Department of Pathology, Hospital Kuala Lumpur, Wilayah Persekutuan, Kuala Lumpur, Malaysia; ^5^Department of Microbiology, Faculty of Biotechnology and Biomolecular Sciences, Universiti Putra Malaysia, Selangor Darul Ehsan, Malaysia; ^6^Malaysia Genome Institute, National Institute of Biotechnology Malaysia, Selangor Darul Ehsan, Malaysia; ^7^Department of Biological Sciences, School of Science and Technology, Sunway University, Selangor Darul Ehsan, Malaysia

## Abstract

The ceramide synthase 2 (*CERS2*) gene has been linked to tumour recurrence and invasion in many different types of cancers including bladder cancer. In this study, the expression levels of *CERS2* in bladder cancer cell lines were analysed using qRT-PCR and the protein expression in clinical bladder cancer histopathological specimens were examined via immunohistochemistry. The potential utility of *CERS2* as a predictive biomarker of response to oncolytic virotherapy was assessed by correlating the *CERS2* mRNA expression to IC_50_ values of cells treated with the Newcastle disease virus (NDV), AF2240 strain. This study demonstrates that *CERS2* is differentially expressed in different types of bladder cancer cell lines and that the siRNA-mediated downregulation of the expression of *CERS2* reduces the migratory potential of UMUC1 bladder cancer cells. However, there were no significant correlations between the expression levels of the CERS2 protein with bladder cancer grade/stage or between the IC50 values of cells treated with NDV and *CERS2* expression. Although the utility of *CERS2* expression may be limited, its potential as an antimigration cancer therapeutic should be further examined.

## 1. Introduction

Bladder cancer is ranked as the ninth most common cancer worldwide, and men are more than three times more likely to develop the disease as compared to women [1, 2]. Due to its high rate of recurrence and invasion, it has the highest cost of treatment per patient as compared to other malignancies [3]. Bladder cancer can be divided into two categories, nonmuscle-invasive bladder cancer and muscle-invasive bladder cancer. Nonmuscle-invasive bladder cancers account for 70% of all bladder cancers and are associated with low risk of progression and metastasis but high risk of recurrence. The treatment of nonmuscle invasive bladder cancer includes transurethral resection followed by a course of chemotherapy or immunotherapy to reduce the recurrence of the tumour [4, 5]. In contrast, muscle-invasive bladder cancers are associated with high rates of progression and metastasis and are usually treated by radical cystectomy if the tumour is organ-confined [4, 6]. To date, there are no prognostic or diagnostic biomarkers as well as predictive biomarkers of therapeutic response that are currently used in the mainstream management of bladder cancer patients [7, 8].

The *CERS2* gene, also known as the tumour metastasis suppressor gene 1 (*TMSG1*), contains Hox-like and TLC domains and is involved in the synthesis of long acyl chain ceramides. This gene was found to be expressed in 12 different types of human tissues, with the highest expression in the liver and kidney and the lowest expression in the colon, spleen, small intestines, thymus, and peripheral blood leukocytes [9]. Increased mRNA levels of the *CERS2* gene were observed in metastatic cell lines as compared to nonmetastatic cell lines, implicating a role for *CERS2* in metastasis [10]. Other studies have identified a correlation between *CERS2* expression with the degree of recurrence and invasion in liver cancer [11], breast cancer [12], cervical cancer [13], pancreatic cancer [14], ovarian cancer [14], prostate cancer [15], and bladder cancer [11]. In a study conducted by Wang et al. [11], bladder cancer patients with low *CERS2*-expressing tumours were shown to have a poorer clinical prognosis.

In recent years, the use of oncolytic viruses such as the Newcastle disease virus (NDV) has become an emerging cancer therapeutic modality. The idea of using NDV in the treatment of cancer has gained vast interest because these viruses are capable of selectively targeting and killing cancer cells [16, 17]. The NDV is a paramyxovirus, round in shape with a bilayer lipid envelope [18]. Oncolysis mediated by NDV occurs through either direct cancer cell killing via the formation of syncytium or indirect cancer cell killing by stimulating an antitumour immune response [19]. To date, no studies have been conducted to determine the potential of *CERS2* as a biomarker that can predict NDV oncolytic response in bladder cancer.

## 2. Materials and Methods

### 2.1. Cell Culture of Bladder Cell Lines

The seventeen bladder cell lines used in this study were purchased from the American Type Culture Collection (ATCC, Manassas, VA, USA) and the European Collection of Authenticated Cell Cultures (ECACC, Salisbury, UK). Cells were cultured and maintained with growth media based on the suppliers' recommendation. All the media were supplemented with fetal bovine serum (FBS) (Thermo Fisher Scientific Inc., Waltham, MA, USA). Cells were incubated at 37°C in a humidified atmosphere of 95% air and 5% CO_2_.

### 2.2. Analysis of *CERS2* Expression in a Panel of Bladder Cancer Cell Lines

RNeasy Mini Kit (Qiagen, Hilden, Germany) was used for extracting the RNA of harvested cells following the manufacturer's protocol. Quantification of the purity and concentration of the RNA was carried out using PCRmax Lambda spectrophotometer (PCRmax, Staffordshire UK). A total of 1 *μ*g RNA was converted to cDNA using QuantiTect® Reverse Transcription Kit (Qiagen, Hilden, Germany). The cDNA synthesis was performed using a thermocycler (Eppendorf, Hamburg, Germany) with the following conditions: incubation for 15 and 3 minutes at 42°C and 95°C, respectively. The quantification of *CERS2* expression levels was then carried out using a miScript SYBR Green PCR Kit (Qiagen, Hilden, Germany) on a LightCycler 480 (Roche, Basel, Switzerland). The qRT-PCR assay was run in triplicates for 45 cycles with the following conditions: preincubation at 95°C and 94°C for 15 minutes and 15 seconds, respectively, followed by amplification for 45 cycles at 55°C, 70°C, and 95°C for 15, 30, and 5 seconds, respectively. Subsequently, the melting phase analysis was conducted from 65°C for 1 minute, followed by a continuous increment to 97°C before finally cooling at 40°C for 30 seconds. The relative expression levels were determined by normalizing to *GAPDH*, *SDHA*, and *TBP* housekeeping genes by using the 2^-*ΔΔ*CT^ method [20].

### 2.3. Knockdown of *CERS2* Expression in UMUC1 Cells

Four small interfering RNA (siRNA) sequences that target *CERS2* knockdown were purchased (Qiagen, Hilden, Germany). The sequences of the siRNAs were as follows: (1) *HS_CERS2_5*—5′ AACCATCGTAAGAATGACTGA 3′ (Cat. no. SI03124198), (2) *HS_CERS2_6*—5′ CATGGCCGTCATTGTGGATAA 3′ (Cat. no. SI04276671), (3) *HS_CERS2_7*—5′ TGCGCTATAGGGTCACTTTAA 3′ (Cat. no. SI04296684), and (4) *HS_CERS2_8*—5′ CCGGCCCAGTCTCCTCAAGAA 3′ (Cat. no. SI04711588). Nonsilencing siRNA with no homology to any known mammalian gene (Cat. no. SI03650318) was used as the negative control (Qiagen, Hilden, Germany) in all knockdown experiments. A total volume of 25 *μ*L of siRNA was added into each well according to the manufacturer's protocol, and the expression of *CERS2* in UMUC1 was determined via qRT-PCR at 24, 48, and 72 hours post-siRNA transfection.

### 2.4. Cell Migration Assay

The migratory potential of UMUC1 cells was measured using a scratch assay. Approximately 60,000 cells were seeded into a 24-well plate. Once the cells reached 100% confluency, 10 g/mL of mitomycin C (Merck, Darmstadt, Germany) was added to the cells and incubated for two hours to stop cell proliferation. A scratch was then made using a 200 *μ*L pipette tip, and the cells were washed with 1x PBS and replenished with fresh media. The cell migration progression was analysed under a microscope. Pictures were captured at three-hour intervals until the gap was closed. The pictures were analysed using the ImageJ software [21].

### 2.5. Immunohistochemical Analysis of *CERS2* Protein Expression in Clinical Bladder Tissues

Sixty-two formalin-fixed paraffin-embedded (FFPE) tissues from bladder cancer patients of various stages and grades were collected from Hospital Kuala Lumpur. The cohort of tissues comprised of 17 Grade 1, 18 Grade 2, and 27 Grade 3 tumours, of which, there were 18 pTa, 19 pT1, 11 pT2, and 14 pT3 and pT4 stages. Tissues were deparaffinized at 36°C for 1 hour. Slides were sequentially rehydrated with 100%, 95%, 80%, and 70% ethanol immersions, followed by antigen retrieval which was done by boiling the slides in sodium citrate using a microwave. The sections were then incubated with a primary polyclonal rabbit antibody (ab85567) against CERS2 (Abcam, Cambridge, United Kingdom) at 4°C overnight and then incubated with Dako DAB chromogen (Dako, Glostrup, Denmark) secondary antibody at room temperature for 10 minutes. A negative control was run in every experiment by replacing the primary antibody with 10% TBS. Sections were finally counterstained using haematoxylin and sequentially dehydrated with 70%, 80%, 95%, and 100% ethanol immersions, mounted with DPX, and then observed under the microscope. Scoring was based on staining intensity.

### 2.6. Correlation between *CERS2* Gene Expression and Sensitivity towards NDV-Mediated Oncolysis

The WST proliferation assay was carried out to determine the cell viability after infection with the NDV AF2240 strain at 5 different concentrations: 0, 1, 3, 5, and 7 HA units using a spectrophotometer at an absorbance of 440 nm. The cell viability test was conducted on 14 cell lines (HTB4, UMUC5, UMUC10, UMUC1, UMUC3, UMUC13, UMUC16, TCCSUP, 5637, SCaBER, RT112, J82, SW780, and HT1376) and calculated at 4 different time points: 24, 48, 72, and 96 hours postinfection. The IC_50_ values were also determined at these four time points. The lower the IC_50_ values, the higher the degree of sensitivity towards NDV-mediated oncolysis. Correlation coefficient (Pearson correlation) analysis was then employed to find the correlation between the expression values and IC_50_ values. The data was analysed and expressed as the correlation coefficient, *R*^2^ (*R*‐squared).

### 2.7. Statistical Analysis

The data in this study were expressed as mean ± standard deviation. Independent *t*-test using GraphPad Prism Version 5.0.3 (GraphPad Inc., US) was employed to determine the significance of the quantitative data obtained. The Kruskal-Wallis test was employed to test the significance of expression of the CERS2 protein across different bladder cancer stages and grades. Results were significant if *p* < 0.05.

## 3. Results

### 3.1. *CERS2* In Vitro Gene Expression

**Figure 1:**
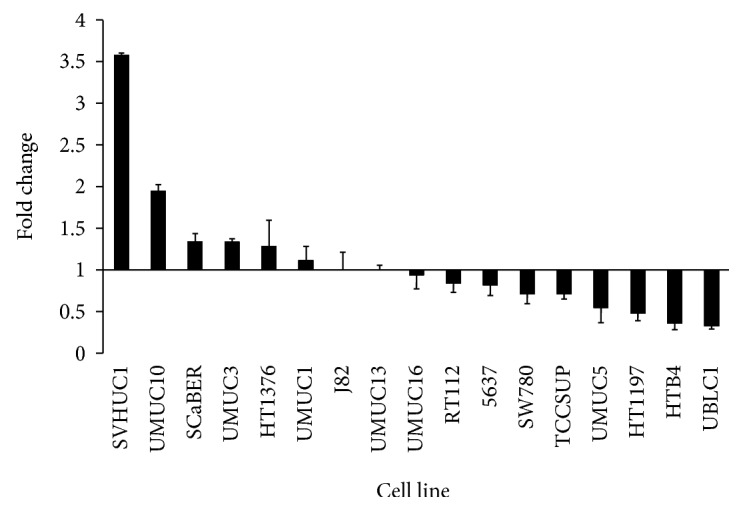
Expression of *CERS2* across different bladder cell lines. The *CERS2* gene expression levels in the 17 bladder cell lines as measured by qRT-PCR, ordered from the highest to lowest expression levels. The fold change for each cell line was calculated relative to the expression of *CERS2* in UMUC13 cells after normalization with the expression levels of housekeeping genes.

The *CERS2* gene expression levels of a panel of bladder cell lines were determined. The cell line with the median level of *CERS2* expression, UMUC13, was selected as the reference cell line. The fold change of *CERS2* expression relative to that of UMUC13 was determined for the remaining cell lines (Figure 1). The SVHUC1 and UMUC10 bladder cell lines were the highest *CERS2-*expressing cell lines with fold changes of 3.58 and 1.95, respectively. Incidentally, SVHUC1 is the only non-cancer cell line included in the study. The cell line was derived from the normal urothelium of the ureter and has been SV40 immortalised. Although it is derived from nonmalignant epithelial cells, 50% of its cells are tetraploid and they harbour various chromosomal aberrations. The lowest *CERS2-*expressing cell lines were UBLC1 and HTB4 with fold changes of 0.33 and 0.36, respectively.

### 3.2. *CERS2* Knockdown In Vitro

**Figure 2:**
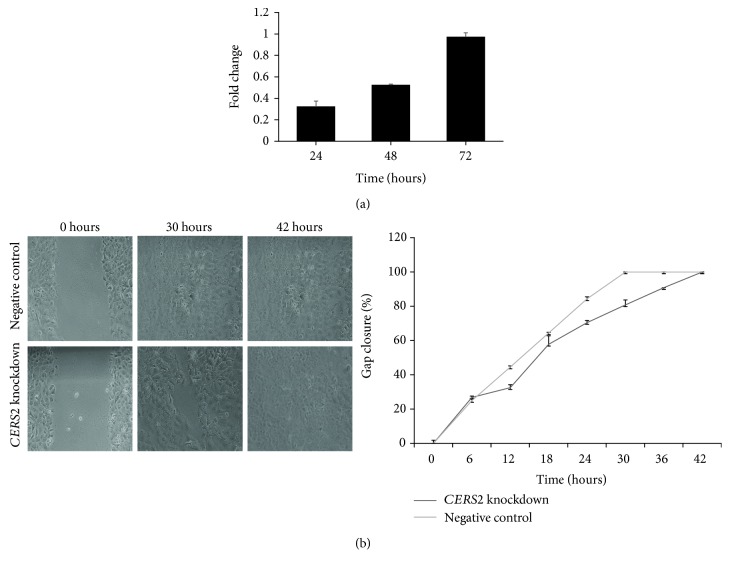
Effect of *CERS2* knockdown on cellular migration in UMUC1 cells. (a) Fold change differences in the expression of *CERS2* between siRNA-mediated *CERS2*-knockdown UMUC1 cells relative to the nontargeting siRNA-transfected negative control at 24, 48, and 72 hours posttransfection. (b) The percentage of gap closure of *CERS2* knockdown versus negative control UMUC1 cells (200x magnification). *CERS2*-knockdown UMUC1 cells have significantly reduced migratory potential after 6 hours postscratch, corresponding to 30 hours post *CERS2*-siRNA transfection.

As compared to the negative control, the levels of the *CERS2* gene were significantly reduced in cells transfected with the combination of 4 *CERS2*-targeting siRNAs. A significant reduction (47.5-67.5%) in the *CERS2* expression was obtained at 24 and 48 hours posttransfection in *CERS2* siRNA-transfected UMUC1 cells relative to the nontargeting siRNA-transfected negative control cells (Figure 2(a)). There was no significant reduction in the expression levels of *CERS2* at 72 hours posttransfection. However, the effect of gene expression knockdown on the protein expression of CERS2 was not evaluated *in vitro*.

### 3.3. Effect of *CERS2* Knockdown on Cell Migration

The scratch assay was employed to investigate the effect of *CERS2* knockdown on UMUC1 cell motility. Images were captured at three-hour intervals from the time the scratch was made until the gap was closed for both *CERS2* siRNA-transfected cells and nontargeting siRNA-transfected negative control cells (Figure 2(b)). The gap in the negative control cells closed within 30 hours, whereas the gap for the *CERS2*-knockdown cells closed within 42 hours postscratch. The rates of gap closure of *CERS2*-knockdown and negative control cells were significantly different (*p* < 0.05).

### 3.4. Analysis of *CERS2* Protein Expression in Histopathological Tissues

**Figure 3:**
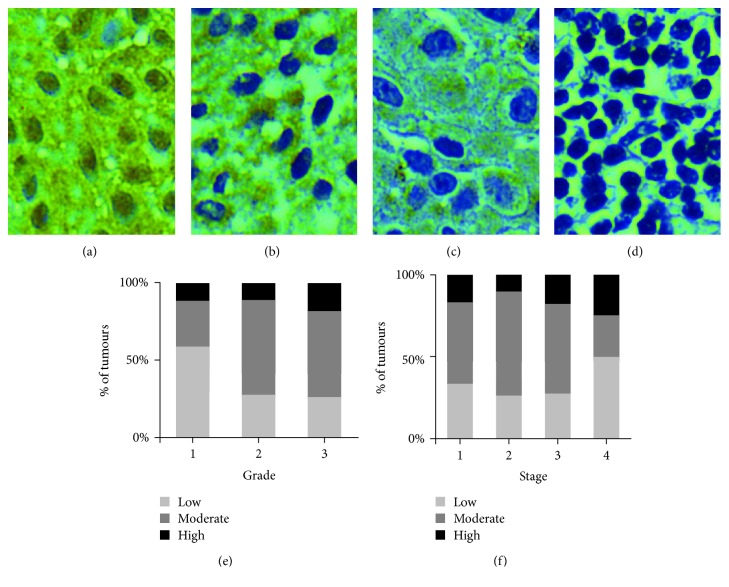
CERS2 protein staining in clinical bladder cancer tissues. Representative cases of (a) strong staining (high expression), (b) moderate staining (moderate expression), (c) weak staining (low expression), and (d) negative control at 400x magnification. The expression of CERS2 in the tissue samples was evaluated based on the staining intensity. CERS2 protein expression in bladder cancer tissues stratified based on tumour grade (e) and stage (f).

Optimal CERS2 antibody concentration was determined by testing the various concentrations of primary antibodies. After a series of optimisation experiments, the optimal antibody dilution was determined to be 1 : 250. All the tissues were scored according to their staining intensities, categorised as weak, moderate, or strong staining (Figure 3). CERS2 staining was mostly localized in the cytoplasm. The majority of Grade 1 tissue samples displayed weak staining intensity, whereas a greater proportion of Grade 2 and 3 tumours had a stronger staining intensity. Interestingly, while a greater proportion of Stage 4 tumours expressed high levels of CERS2 as compared to other tumour stages, there was also a comparatively greater percentage of Stage 4 tumours that expressed low levels of CERS2. This was because there were a smaller proportion of Stage 4 tumours that displayed moderate expression of CERS2. Nevertheless, no statistical significant differences in CERS2 protein expression across different tumour stages and grades were identified in this study (Figures 3(e) and 3(f)).

### 3.5. Correlation Analysis between *CERS2* Gene Expression and NDV-Mediated Oncolysis

**Figure 4:**
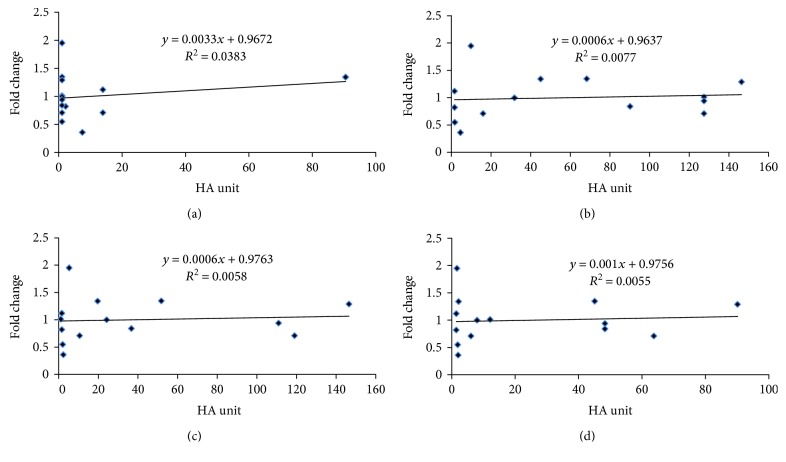
Correlation between *CERS2* gene expression and sensitivity to NDV-mediated oncolysis. The series of graphs show the correlation between IC_50_ values and *CERS2* expression across 14 cell lines at (a) 24 hours, (b) 48 hours, (c) 72 hours, and (d) 96 hours post-NDV infection. The correlation efficiencies are presented as *R*^2^ values.

The correlation analysis was done to determine whether the expression of *CERS2* was associated with bladder cancer cellular sensitivity towards NDV-mediated oncolysis. The correlation coefficient *R*^2^ values were 0.0383 at 24 hours postinfection (*r* = 0.19; Figure 4(a)), 0.0077 at 48 hours postinfection (*r* = 0.087; Figure 4(b)), 0.0058 at 72 hours postinfection (*r* = 0.076; Figure 4(c)), and 0.0055 at 96 hours postinfection (*r* = 0.074; Figure 4(d)). All of the *R*^2^ values obtained indicate that a weak correlation exists between the IC_50_ values and *CERS2* gene expression levels across the four different time points.

## 4. Discussion

**Figure 5:**
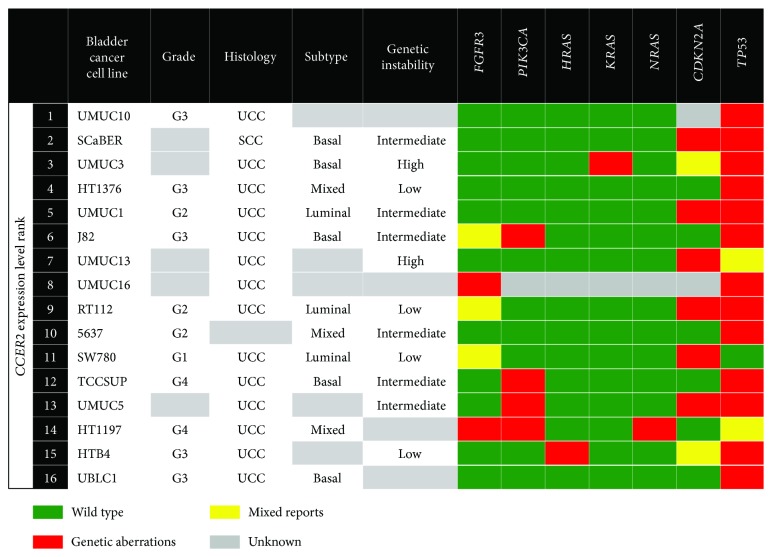
Molecular and histological characteristics of bladder cancer cell lines ranked based on *CERS2* expression levels (highest expression (rank 1) to lowest expression (rank 16)). Source of molecular and histological characteristics: [23–25].

The *CERS2* gene was found to be differentially expressed across the different bladder cancer cell lines that were investigated in this study. A study conducted by Zhao et al. [22] found that the lower the expression levels of *CERS2*, the more aggressive the bladder cancer cell lines were, suggesting that *CERS2* expression correlates with bladder cancer progression and metastasis. The cell lines used in this study were ranked based on the level of *CERS2* expression and mapped to the histology, molecular subtype, genetic instability, and mutational status of classical genes associated with bladder cancer as determined and catalogued in previous studies [23–25] (Figure 5). The only squamous cell carcinoma cell line in this study, SCaBER, was the 2^nd^ highest *CERS2*-expressing cell line. There were no obvious distinctions between high- and low-*CERS2*-expressing cell lines in terms of the grade of the primary tumour, molecular subtype (basal, mixed, and luminal) and genetic stability. Interestingly, the five highest *CERS2*-expressing cell lines harboured lower frequency of *FGFR3* and *PIK3CA* mutations as compared to the lower *CERS2*-expressing cell lines. The differential *CERS2* expression across the various bladder cancer cell lines in this study reflects tumour heterogeneity and that the functional significance of *CERS2* expression may vary across different bladder tumours.

To investigate the metastasis-suppressing potential function of *CERS2*, the expression of *CERS2* was downregulated through siRNA-mediated knockdown. *CERS2* knockdown was established in UMUC1 cells instead of the higher *CERS2*-expressing bladder cancer cell lines (UMUC10, SCaBER, UMUC3, and HT1376) because these cells were growing at a very slow rate. The *CERS2* expression was downregulated by 47.5% at 24 hours and 67.5% at 48 hours posttransfection. The downregulation of *CERS2* expression reduced the migratory potential of UMUC1 cells. These findings are in contrast to the findings by Wang et al. [26], where they found that the migratory potential of RT4 and T24 bladder cancer cell lines increased upon siRNA-mediated knockdown of *CERS2* expression. The opposing roles that *CERS2* plays in different types of bladder cancer cells suggest that the influence of *CERS2* on the migratory potential phenotype is dependent on other factors such as the combinatorial expression of other genes.

For instance, although *CERS2* is involved in the production of ceramide that in turn inhibits the migration, proliferation, and survival of cancer cells [27], ceramide can be metabolized to form sphingosine 1-phosphate (S1P), an antiapoptotic agent that increases cancer cell migration and proliferation as well as survival [28]. The UMUC1 cells that express *CERS2* potentially produce ceramide that is subsequently metabolized into S1P. Thus, the knockdown of *CERS2* may have led to the lowering of ceramide levels, resulting in the reduction of S1P production that in turn caused the inhibition of the migratory potential of the UMUC1 *CERS2*-knockdown cells. Another reason for the decreased migratory potential in the *CERS2*-knockdown cells could be explained through another study in neuroblastoma [29]. Findings from the study suggest that *CERS2* knockdown activates the unfolded protein response and induces autophagy, thus decreasing the migratory rate of transfected cells as observed in our study.

In this study, the mechanism in which *CERS2* knockdown exerts an antimigratory effect on UMUC1 bladder cancer cells is not established, but while many studies suggest *CERS2* as a putative pan-cancer metastatic suppressor gene, there are studies that suggest a more protumourigenic role for *CERS2*, albeit to a lesser extent [12, 30]. The study conducted on breast cancer tissues by Schiffmann et al. [12] revealed a 12-fold increase in the levels of *CERS2* in malignant tissues as compared to normal tissues, while the study conducted by Erez-Roman et al. [30] showed that *CERS2* was highly expressed in about half of the invasive ductal carcinoma patients examined. The duality of genes that harbour functions as oncogenes and tumour suppressor genes, manifesting differently in different tumours, have been identified in many other protumourigenic genes with tumour-suppressive function ([31]). Thus, further studies need to be conducted to effectively delineate the context in which the level of *CERS2* affects bladder cancer cell phenotype.

To investigate the potential role of *CERS2* as a biomarker for bladder cancer, immunohistochemistry analysis was conducted to evaluate the patterns of expression in bladder cancer histopathological tissues. Although a greater proportion of high grade and stage tumours expressed high levels of CERS2 protein, there were no statistically significant differences in the expression levels across different grades and stages. This is contradictory to the findings from a previous study conducted by Wang et al. [11] that observed an association with the depth of invasion. A possible explanation for such discrepancy may be due to the small number of tissue samples screened in this study or the heterogeneous nature of the disease.

Previous studies have demonstrated the oncolytic activity of the NDV AF2240 strain as an anticancer agent against cancer cells [32]. An inverse correlation between antiviral gene expression and NDV susceptibility in several cell types has been identified [33]. This study found a weak correlation between *CERS2* expression and IC_50_ values of NDV-mediated oncolysis. The weak correlation suggests that *CERS2* expression is not a suitable biomarker for predicting bladder cancer response towards NDV anticancer therapy. However, further investigation into the interactions between the *CERS2* gene and other genes within pathways related to the responses to NDV therapy may provide insights into identifying more promising biomarkers.

## 5. Conclusion

Findings from our study revealed that the *CERS2* gene is differentially expressed in different types of bladder cancer cell lines. It was demonstrated that the knockdown of the *CERS2* gene resulted in the reduction of the migratory potential of UMUC1 cells. The CERS2 protein was differentially expressed in the bladder cancer tissues analysed, where some tumours expressed high levels while some tumours expressed none or low levels of CERS2. However, there was no statistically significant association between the grade or stage and the intensity of staining. Finally, there was no correlation between the expression of *CERS2* in bladder cancer cell lines and their sensitivity towards NDV-mediated oncolysis. The promigratory role of *CERS2* identified in this study that contrasts previous studies in different bladder cancer cells should be further investigated to clarify the context in which the expression of *CERS2* modulates the cancer cell migration phenotype.

## Data Availability

The data used to support the findings of this study are available from the corresponding author upon request.
